# 25, 50 & 75 years ago

**DOI:** 10.1111/ans.18176

**Published:** 2022-11-24

**Authors:** Julian A. Smith

**Affiliations:** ^1^ Department of Surgery Monash University Melbourne Victoria Australia

## Twenty‐five years ago

**Iannuzzi A, Khadra M, Boulas J. Renal parenchyma‐sparing surgery in carcinoma. *ANZ J. Surg*. 1997; 67:854–6.**

There is controversy regarding the role of renal‐sparing surgery in patients with kidney cancer who have a functioning contralateral kidney. The present study aimed to review the recent experience of renal‐sparing surgery at Royal Prince Alfred Hospital (RPAH), Sydney. Eighteen consecutive patients undergoing conservative surgery for kidney tumours at RPAH between February 1987 and January 1995, were reviewed. Eleven patients had imperative indications for conservative surgery and the remaining seven patients had elective indications. Ten patients had modified enucleation with a margin of normal parenchyma. Six patients underwent partial nephrectomy and two had wedge resections. Patients were followed up at 1, 6 and 12 months, and thence every 6–12 months. Follow‐up ranged from 9 to 104 months (mean: 46.2 months, median: 48 months). Sixteen of the 18 patients were still alive at the end of the follow‐up (October 1995), with no clinical evidence of local or distant metastasis. The two deaths were not related to the fact that these patients had conservative surgery. The average tumour dimensions were 43 mm × 49 mm, with an average volume of 194 mm^3^. All resections were complete, with margins ranging between 1.0 and 20.0 mm (mean: 8.7 mm). The survival rate in the present study is comparable to those found by other researchers. Conservative surgery is indicated in renal tumours where radical surgery would render the patient anephric. Conservative surgery, however, is controversial in a patient with a normal contralateral kidney. The present study has shown that renal parenchyma‐preserving surgery for localized tumours provides a feasible treatment option.

**Cohen RJ, Robinson E, McRae CU. Prostate cancer mortality in New Zealand: the past and projections for the future. *ANZ J. Surg*. 1997; 67:781–4.**

Ageing male populations and improved diagnosis of early stage disease have contributed to the increasing incidence of prostate cancer observed in many Western countries. The clinical significance of these diagnosed cancers is, however, currently unclear. The aim of this study is to examine trends over time in prostate cancer mortality as an indicative measure of clinically significant disease during a 29‐year period (1965–1993) which preceded the extensive use of early cancer diagnostic techniques or radical therapy protocols. Age‐specific and age‐standardized rates were calculated for each year during the study period, using routinely collected mortality and demographic data. A Poisson regression model was used to describe trends in the age‐specific rates over time to predict numbers of prostate cancer deaths and the lifetime risk of death over the next 20 years. Significant annual increases ranging from 1% to 2.6% were found for age‐specific prostate cancer mortality rates over the 29‐year time period, with the largest increases experienced in the younger age groups at risk. Based on projected population ageing and growth alone, annual numbers of prostate cancer deaths are predicted to increase from 487 in 1993 to 664 by the year 2006 and then to 833 by the year 2016. Continuation of the observed increases in age‐specific mortality rates would result in a predicted 797 deaths by the year 2006, while an expected 1115 deaths is calculated for the year 2016. This would correspond with an increase in the lifetime risk of death from prostate cancer from a present 3.7% to 4.5% in 10 years and 6.2% in 20 years. The changing pattern of prostate cancer mortality described in this study is likely to represent a significant increase in the incidence of clinically significant disease. This will have a significant impact on the ageing New Zealand male population, and important implications for the provision of effective treatment and preventive strategies.

## Fifty years ago

**Martin LW. A different approach permitting portal‐systemic shunt for extrahepatic portal thrombosis. *ANZ J. Surg*. 1972; 42:123–5.**

Studies of the anatomy and pathology in children with extrahepatic portal thrombosis and portal hypertension have shown that the portal vein is generally free of thrombosis for a distance of 1.5 to 2 cm beyond the junction of the superior mesenteric with the splenic vein. In nine children, it has been possible to dissect out this stump of portal vein and anastomose it directly in an end‐to‐side fashion to either the vena cava or to the central portion of the left renal vein where it crosses anterior to the aorta. The spleen is not removed. The operation can be employed successfully following a previous splenectomy or following a previous unsuccessful conventional splenorenal shunt. Follow‐up evaluation has been gratifying, with apparent relief of portal hypertension in each instance.

**Balasegaram M. Hepatic surgery: a review of a personal series of 95 major resections. *ANZ J. Surg*. 1972; 42:1–10.**

This article briefly reveals the author's experience of 95 major hepatic resections performed for various conditions during a 7‐year period. The necessity for better understanding of intrahepatic anatomy of the liver, especially of the hepatic venous anomalies, and the use of more recent diagnostic aids, is stressed. The author's indications and the types of resection performed are described in detail. Some of the views expressed are in direct opposition to the traditional methods of treatment of some hepatic lesions. The low mortality of 12.6% in this series has been attributed to proper selection of patients for resection, and advancements in anaesthesia and surgical technique, including better preoperative and postoperative care.

## Seventy‐five years ago

**Kinsella VJ. A clamp for crushing the colostomy spur. *ANZ J. Surg*. 1947; 16:283–4.**

**A new clamp–a long‐handled lever of the third order.**

**Fig. 1:**
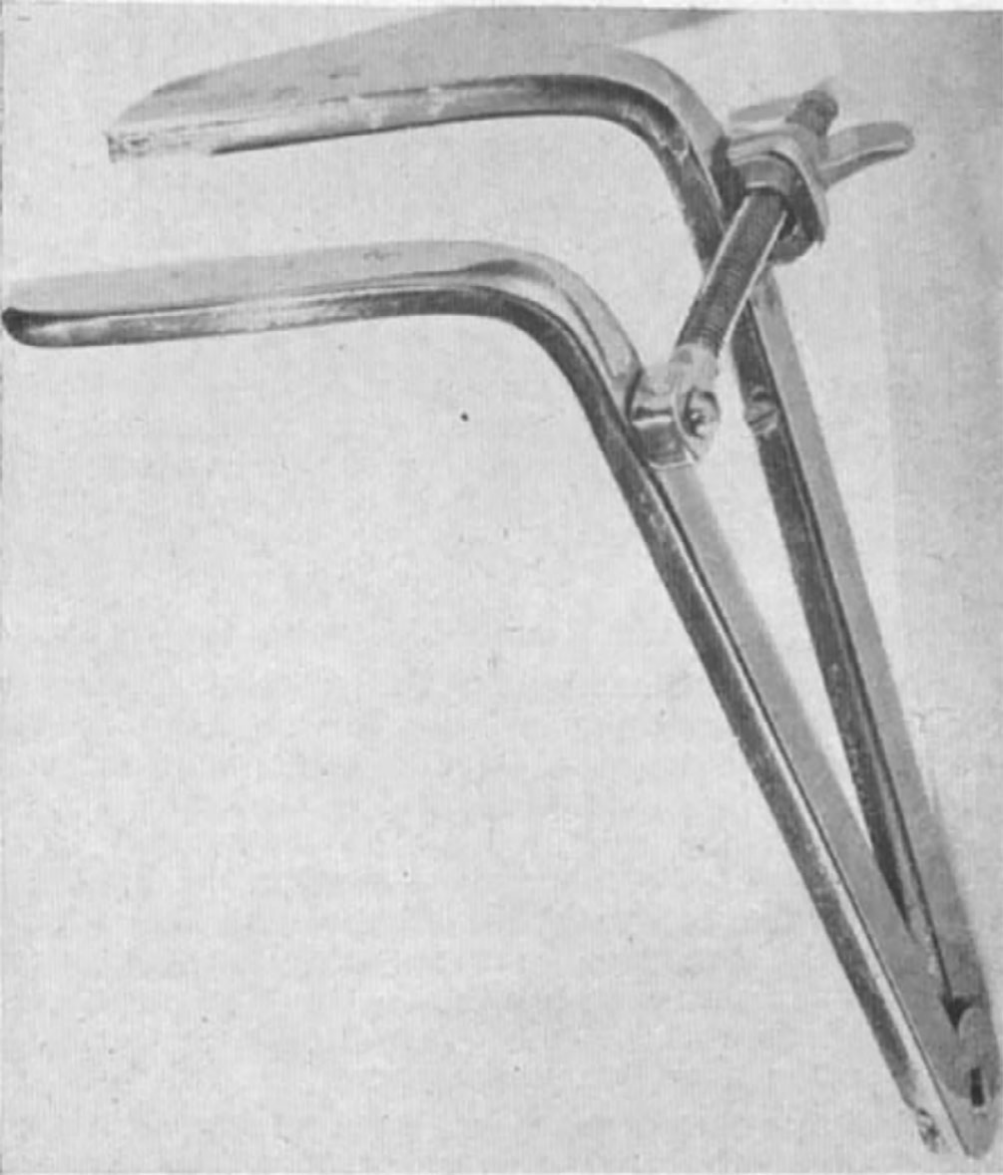
Colostomy spur‐crusher.

**Fig. 2:**
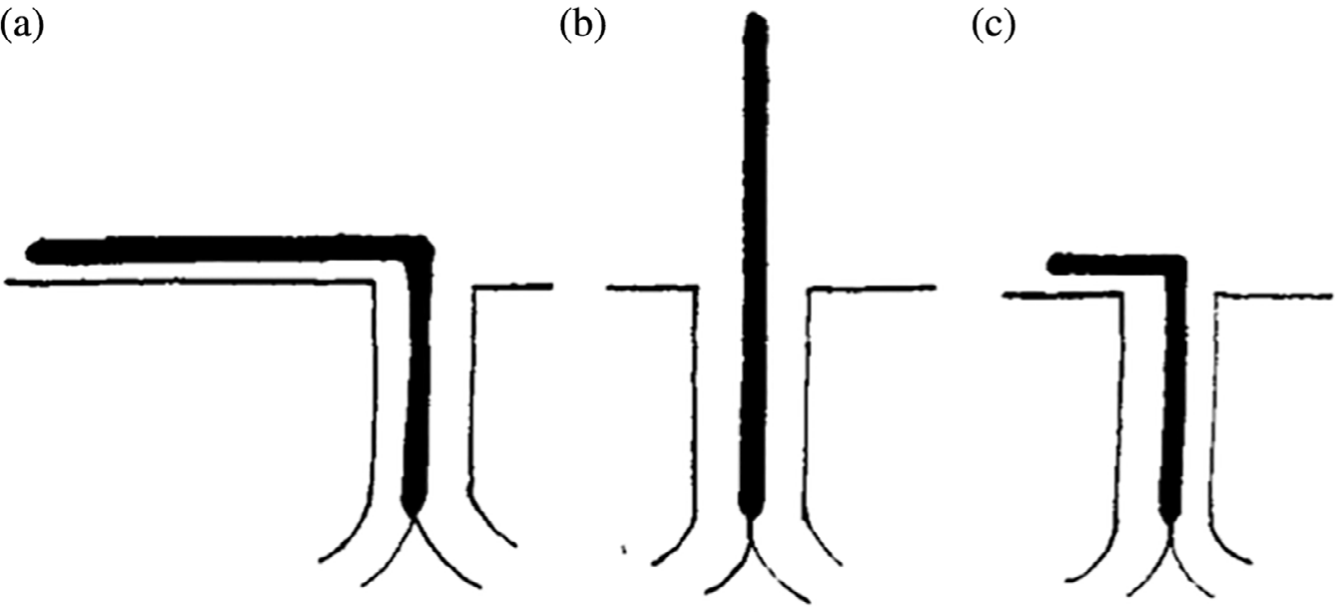
Stabilizing the clamp. The figure shows the advantage of the right‐angled clamp with the long handle (a) over the straight clamp (b) and over the right‐angled clamp with a short handle (c).

A new clamp, made of stainless steel, is a lever of the third order (Fig. 1). The length from hinge to thumb‐screw (the handle) is greater than the length from thumb‐screw to tip (the blades) 140 cm as compared with 90 cm. Therefore, the lever is mechanically efficient. The blades are bent at right angles to the handle, remain parallel when the instrument is opened, and can be easily inserted. The bending of the instrument enables the handle to rest snugly upon the wall of the abdomen. The length of the handles and their flattened shape facilitate secure fixation. This must be emphasized as essential in a spur‐crushing clamp. It prevents visceral trauma by rocking or rotation of the instrument, induced by the patient's movements. Figure 2 shows clearly the advantages in this respect of the right‐angled clamp with long handles over the other types. A long, narrow pad is placed under the handle of the clamp, and its edges are folded round the handle so as to leave skin space for strapping. The handle is then firmly fixed by broad strapping. A thick pad is then placed on the abdomen and a binder is applied. A non‐residue diet is prescribed. The patient can move and turn, even on to the side of the clamp, and sit up for meals, with an ease and comfort surprising to the surgeon who has had experience with the older types of clamp.

